# Effects of 30 days of ketogenic diet on body composition, muscle strength, muscle area, metabolism, and performance in semi-professional soccer players

**DOI:** 10.1186/s12970-021-00459-9

**Published:** 2021-09-16

**Authors:** A. Antonio Paoli, Laura Mancin, Massimiliano Caprio, Elena Monti, Marco V. Narici, Lorenzo Cenci, Fabio Piccini, Matteo Pincella, Davide Grigoletto, Giuseppe Marcolin

**Affiliations:** 1 Department of Biomedical Sciences, University of Padua, Via Marzolo, 3, 35131 Padua, Italy; 2 Research Center for High Performance Sport, UCAM, Catholic University of Murcia, 30107 Murcia, Spain; 3 Human Inspired Technology Research Center, University of Padua, Padua, Italy; 4 Department of Human Sciences and Promotion of the Quality of Life, San Raffaele Roma Open University, Rome, Italy; 5 Italian Microbiome Project, Milan, Italy; 6 Inter FC., Milan, Italy; 7 FIGC Federazione Italiana Giuoco Calcio (Italian Football Federation), Rome, Italy

**Keywords:** Ketogenic diet, Soccer, Body composition, Yo-yo intermittent test, Muscle cross sectional area, Metabolism

## Abstract

**Background:**

A ketogenic diet (KD) is a nutritional approach, usually adopted for weight loss, that restricts daily carbohydrates under 30 g/day. KD showed contradictory results on sport performance, whilst no data are available on team sports. We sought to investigate the influence of a KD on different parameters in semi-professional soccer players.

**Methods:**

Subjects were randomly assigned to a iso-protein (1.8 g/Kg body weight/day) ketogenic diet (KD) or western diet (WD) for 30 days. Body weight and body composition, resting energy expenditure (REE), respiratory exchange ratio (RER), cross sectional area (CSA) and isometric muscle strength of quadriceps, counter movement jump (CMJ) and yoyo intermittent recovery test time were measured.

**Results:**

There was a significantly higher decrease of body fat (*p* = 0.0359), visceral adipose tissue (VAT) (*p* = 0.0018), waist circumference (*p* = 0.0185) and extra-cellular water (*p* = 0.0060) in KD compared to WD group. Lean soft tissue, quadriceps muscle area, maximal strength and REE showed no changes in both groups. RER decreased significantly in KD (*p* = 0.0008). Yo-yo intermittent test improved significantly (*p* < 0.0001) in both groups without significant differences between groups. CMJ significantly improved (*p* = 0.0021) only in KD.

**Conclusions:**

This is the first study investigating the effects of a KD on semi-professional soccer players. In our study KD athletes lost fat mass without any detrimental effects on strength, power and muscle mass. When the goal is a rapid weight reduction in such athletes, the use of a KD should be taken into account.

**Trial registration:**

registered retrospectively on Clinical Trial registration number NCT04078971.

## Introduction

Soccer is one of the most popular and well-known sport all over the world [1] and lots of factors need to be considered when handling with elite soccer players [2]. Technical skills, game intelligence, proper mindset, athletic characteristics, general fitness condition and body composition represent essentials features in the make-up of a soccer player. Elite players show excellent values of body composition [3] while semi-professionals usually tended to have worst findings. In Italy, Elites amount to more than 2500 while semi-professionals add up to about 377,000 [4]. The excess of body weight and body fat, lower lean soft tissue, fluid and electrolyte imbalance, is related to detrimental effects upon soccer health and soccer-specific actions such as dribbling, ball control, speed and power [5], thus, for semi professional athletes, who usually perform soccer in their leisure time, a good body composition is recommended even though hard to achieve. To get adequate body composition and maintaining an excellent general health, athletes need to consider several aspects ranging from a correct training program to proper sleep and recovering approach; however, one of the most influencing aspect of body composition is diet [6]. Different nutritional approaches have been used according to specific player’s characteristics, eating habits and different energy demands during competitive or non-competitive season [7]. The up to date dietary approaches are also focused on enhancing recovery and preventing injuries by providing antioxidants, vitamins, polyunsaturated fatty acids and collagen [8]. During competitive season, a relatively high carbohydrate diet is usually recommended both on training days and matches [9]. During off-season, semiprofessional players often show an increase of body weight and body fat; in such case it is important to avoid detrimental strategies that are usually recommended before the beginning of in-season [10]. These methods, such as extreme caloric restriction, are deleterious both for health and athletic performance outcomes [11]. As opposed to the drastic energy restriction approaches, the ketogenic diet (KD) is a nutritional strategy consisting of high fat, adequate protein and low carbohydrate intake (less than 5% of total daily energy intake or 30 g of carbohydrate (CHO) daily)), in which the amount of total available energy is adequate [12]. Hans Krebs referred to the metabolic state of diet-induced ketosis as “physiological ketosis” to distinguish it from the pathological diabetic ketosis [13]. The physiological function of ketosis is to sustain muscle works and central nervous system functions during reduced glucose availability with the high energy metabolic substrate of ketone bodies (KBs), which are small molecules produced by a process called ketogenesis from fats; by this mechanism ketones allowed ancestors to survive and remain efficient even though deprived of glucose [14]. The ketone bodies (acetoacetate, acetone, and β-hydroxybutyrate) are produced in the liver under low-carbohydrate availability conditions, acting as an alternative energy source for peripheral tissue, such as skeletal muscle, brain and heart [15] . There are conflicting data about the use of KD in sports, some researches showed negative effects on performance [16–18] whilst others suggest instead a positive effect or, at least, no detrimental effects [19–23]. As a matter of fact, the use of KD in sports may have different aims if we consider endurance or strength/power athletes [12]. To the best of our knowledge, no data are available about the effects of KD in team sports. Our hypothesis is that 30 days of KD may be able to maintain muscle mass without affecting specific performance [19, 24], while reducing body fat in a model of mixed endurance/power sport such as soccer. Given the above, the purpose of our study was to determine the effects of thirty-days of KD on body composition, muscle strength, muscle area, metabolism and performance in semi-professional soccer players.

## Material and methods

### Participants

Sixteen semi-professional male soccer players (age 25.5 ± 2.8 years; height 179.0 ± 9.2 cm; body mass 77.2 ± 11.88) who competed in a local team (A.S.D. Riviera Del Brenta, Venezia, category one), were recruited to participate in this study (anthropometric baseline characteristics of subjects are shown in Table 1). Exclusion criteria included a body fat percentage over 32%, (determined via dual energy X-ray absorptiometry DXA), cardiovascular, respiratory, gastrointestinal, thyroid or any other metabolic diseases, weight change ±2 Kg over the last month, adherence to special diets, use of nutritional supplements (except a daily multivitamin-mineral) and use of medication to control blood lipids or glucose and goal-keepers. During the study protocol players were asked to keep their normal and constant training schedule (8 h of training/week) during the study period. All the subjects read and signed the informed consent document with the description of the testing procedures approved by the ethical committee of the Department of Biomedical Sciences, University of Padova, and conformed to standards for the use of human subjects in research as outlined in the Declaration of Helsinki, Clinical Trial registration number NCT04078971.

**Table 1:** Baseline characteristics of athletes

	KD ***n*** = 8	WD ***n*** = 8
Age (years)	25.5 ± 2.5	25.5 ± 3.1
Height (cm)	178.7 ± 8.6	179.4 ± 9.8
Body Mass (Kg)	78.19 ± 11.7	76.15 ± 12.0
DXA Body Fat (Kg) DXA	19.475 ± 4.0	18.88 ± 6.6
DXA Body Fat (%) DXA	24.78 ± 3.5	25.03 ± 4.4
BMI (Kg/m^2^)	24.5 ± 2.1	25.4 ± 2.6
Lean Mass (Kg)	57.4 ± 4.5	56.6 ± 5.1

### Study design and procedures

The study was as a randomized, parallel arm, controlled, prospective study. Subjects underwent different measurements in three different consecutive days at the beginning of the study and after 30 days. Measurements were taken by the same operators and in the same conditions.

Subject were randomly assigned to a very low carbohydrate ketogenic diet (KD *n* = 8) group or Western Diet (WD *n =* 8) group, through an online computer generated sequence (www.graphpad.

com/quickcalcs/randomN1.cfm), matched for percentage of body fat.

**Fig. 1:**
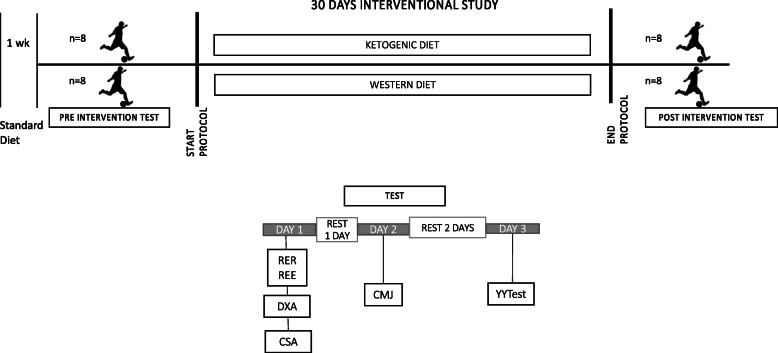
Measurements performed in three different following days. Before and after 30 days. REE: resting energy expenditure; RER: respiratory exchange ratio; DXA: dual-energy X-ray absorptiometry; CSA: cross sectional area; MCV: maximal voluntary isometric contraction; CMJ: counter movement jump; Yo-yo test: yo-yo intermittent recovery test level 1

The work load of all athletes was over-imposable because the coach and trainers strictly controlled the training schedule and they were instructed to maintain the same level of physical activity throughout the study. (The study protocol is shown in Fig. 1)*.* Before the start of the experiment a meeting was scheduled in order to advise participants about the protocol of study and to give them the first useful and needed advices.

### Dietary intervention

**Fig. 2:**
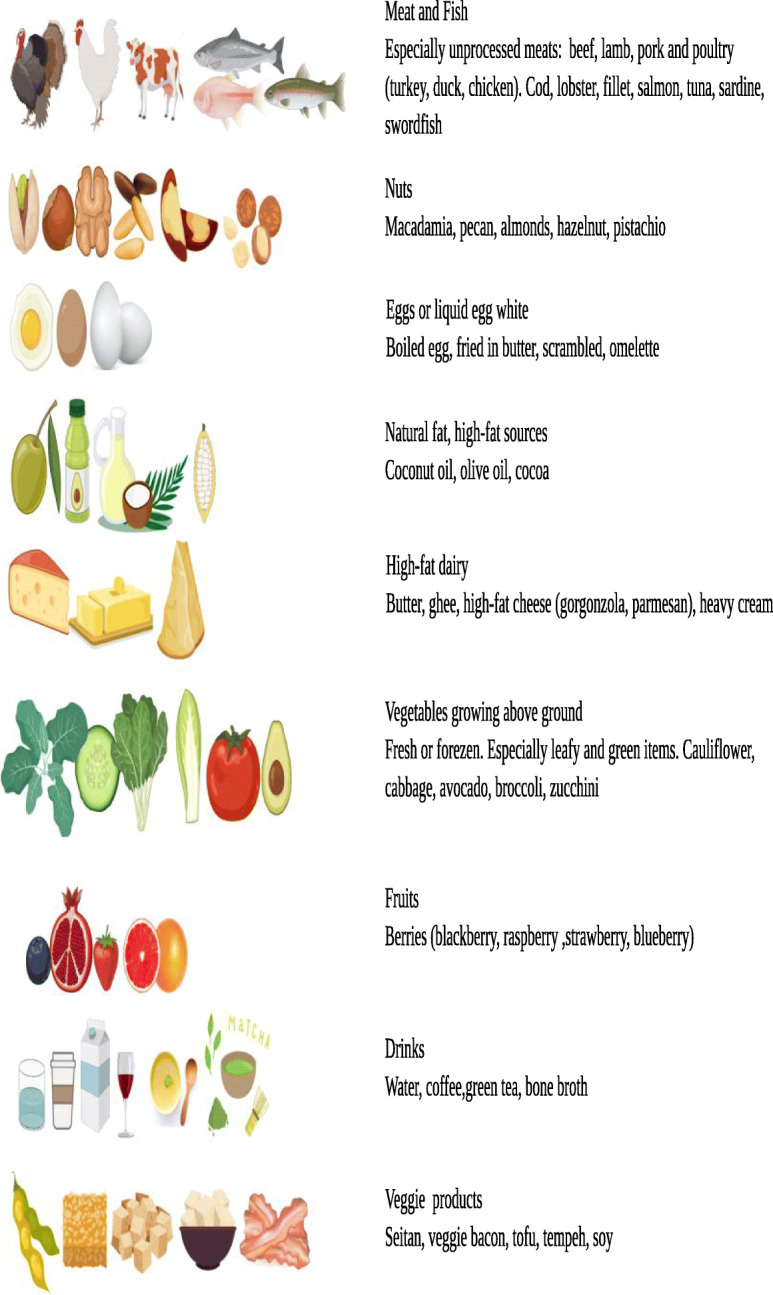
List of food allowed during the ketogenic (created with BioRender.com)

Before the start of the study, athletes were provided nutritional counseling and resources to better adhere to KD. Resources included food lists containing the food prohibited and permitted in ketogenic diet and electronic-suggested daily meal plans, meal recipes. The food lists encouraged the consumption of beef, veal, poultry, fish, raw and cooked vegetables without restriction, cold cuts such as dried beef, eggs and seasoned cheese (parmesan), Konjac, fruits with the lowest glycemic index (blueberry, raspberry), raw nuts and seeds, ghee butter, plant oils and fats from avocado, coconut and olives ^31^. The drinks permitted were tea, coffee, herbal extracts without sugar and a “*Keto cocktail*” was allowed once per week, made up of gin and soda. The foods and drinks to be avoided were alcohol, bread, pasta, rice, milk, soluble tea and potatoes (a detailed list is provided in Fig. 2). A nutritional protocol as the KD may be hard to be maintained for long periods due to the lack of sweet taste [25], thus, for this reason, in the last years, many ready-to-eat ketogenic products (RKP) have been created [26] in addition to usual low carbohydrate foods [27]. In our protocol we used some RKP as a ketogenic pasta (selected with a ketogenic ratio of fats: protein+carbohydrate equal to 4:1) (Le Gamberi Foods, Forlì, Italy), and other RKP (specialty meals and drinks) that mimics the taste of carbohydrates [27], constituted principally of high-quality protein (18 g of protein per portion), fibers, and electrolytes (mainly magnesium and potassium) [28] (Tisanoreica® by Gianluca Mech S.p.A., Asigliano Veneto, Vicenza, Italy). It has been demonstrated that the use of RKP increases the adherence to the ketogenic nutritional protocol [25, 29, 30]. Both diets were designed to be isoproteic (1.8 g x Kg^− 1^ x body weight^− 1^ x day ^− 1^). The distribution of macronutrients during the very low carbohydrate ketogenic diet (KD) was: carbohydrate (< 30 g x day^− 1^; < 10%) protein 1.8 g x Kg^− 1^ x body weight^− 1^ x day ^− 1^ (~ 25–30%), fats (~ 65–70%)*.* Moreover each subject was provided with three herbal extracts (Table 2) according to commercial ketogenic protocol (Tisanoreica®, Gianluca Mech S.p.A., Asigliano Veneto, Vicenza, Italy). During the first week, subjects were provided with pure medium chain triglyceride oil (MCT oil: 20 g Named® Natural Medicine), in order to facilitate ketosis [31] and to allow players maintaining the same work load during training sessions. WD group was provided with a diet similar to western diet, thus the intake of protein has been increased to 1.8 g x Kg^− 1^ x body weight^− 1^ x day^− 1^ in order to be isoproteic. The WD was composed mainly of whole cereals (spelt, rye, oat) and pseudo-cereals (buckwheat, quinoa, amaranth), whole grain pasta, potatoes, meet, fish, vegetables, fruit, legumes, olive oil, milk and red wine (at most 1 glass per day). The WD was composed to ensure a constant energy and macronutrient balance: protein 1.8 g x Kg^− 1^ x body weight^− 1^ x day^− 1^, (~ 30%), fats ~ 20–25% and carbohydrate ~ 50–55%. WD diet was also designed to contain < 10% saturated fat and < 300 mg cholesterol/day. In both groups, protein intake has been well drafted and divided throughout the day. Protein intake was distributed equally throughout the day (every 3–4 h). Pre-sleep casein protein intake (30–40 g) was provided in both group after training evening session, as indicated by ISSN [32]. In addition, it has been recommended to athletes to drink an adequate intake fluids during the day (~ 1500-2000 mL), especially before and after training sessions and match (300-500 mL before and 500 mL after training). The diets were explained to all subjects during an individual visit and dietary intake was measured by validated 3-food-diary that has been used in the past in studies with athletes [33] and analyzed by *Nutritionist Pro™ (AxxyA systems, Arlington, VA).* Subjects received thorough instruction for completing detailed weighed food records during 7 day-periods for each diet. Food measuring utensils and scales were provided to subjects to ensure an accurate reporting of foods and beverages amounts consumed. To ensure that carbohydrates were restricted throughout the KD diet, subjects tested their urine daily using reagent strips at the same time of the day (Ketostix semiquantitative urine strips, Bayer, Leverkusen, Germany), recording the result on log sheet and, once or twice a week subjects were tested by *GlucoMen LX Plus (Menarini Diagnostics, Firenze, Italy)* to detect ketones concentration in capillary blood. Subjects received follow-up counselling and dietetic education if necessary. A *whatsApp* (Facebook Inc., Mountain View, CA, USA) group was created and some applications for smartphone were provided (*Keto-diet tracker,* *https://ke.to*; Keto-app*,* *https://ketodietapp.com**),* in order to track their food daily intake. Moreover, the nutritionist contacted each participant weekly to ensure a proper dietary adherence. While the WD group received nutritional guidelines on how to formulate a WD diet according to their daily energy requirements, the KD group’s suggestion included information on how to formulate a KD diet and a more accurate “shopping list and example meal plans”. Both KD and WD subjects were followed by an app to verify the adherence to diet.

**Table 2:** Plant extracts and composition

Plant extracts	***Composition***
Extracts 1, ml/day	Durvillea antarctica, black radish, mint, liquorice, artichoke, horsetail, burdock, dandelion, rhubarb, gentian, lemon balm, chinaroot, juniper, spear grass, elder, fucus, anise, parsley, bearberry, horehound
Extracts 2, ml/day	Horsetail, asparagus, birch, cypress, couch grass, corn, dandelion, grape, fennel, elder, rosehip, anise
Extracts 3, ml/day	Eleuthero, eurycoma longifolia, ginseng, corn, miura puama, grape, guaranà, arabic coffee, ginger
Extracts 4, ml/day	*L. usitatissimum* L., Gelidium amansii, Rheum officinalis L., Cynara scolymus L., Matricaria chamomilla L., Gentiana lutea L., Mentha piperita L., Pimpinella anisum L., Glycyrrhiza glabra L., Raphanus sativus L., Foeniculum vulgare Mill., *A. officinalis* L., Melissa officinalis L., Juniperus communis L.

### Body composition

Body weight was measured to the nearest 0.1 Kg using an electronic scale (Tanita BC-545 N Amsterdam, Netherlands), and height to the nearest 1 cm using a wall-mounted stadiometer (GIMA S.p.a., Milan, Italy). Whole body and regional body composition were measured in the morning after a 12 h overnight fast by dual energy X-ray absorptiometry (DEXA, Hologic Horizon TM QDR RSeries Bedford, Massachusetts, USA) (fat mass: ICC = 0.995, SEM =0.81 Kg; lean soft tissue: ICC = 0.995, SEM = 0.83 Kg). Regional analysis of body composition, trunk and visceral adipose tissue (VAT) were calculated according to anatomical landmarks by the same technician using computer algorithms (software APEX 3.0, Hologic Bedford, Massachusetts, USA) (ICC = 0.99, SEM = 7.7 g). All scans were performed by a qualified physician. Calibration of the densitometer was checked daily against standard calibration block supplied by the company (Phantom 21, 965 Lumbar spine with characteristics of 4 hydroxyapatite vertebrae included in resin. Coefficent of Variation: 0.415%). Extra cellular water has been measured by bioelectrical impedance analysis (BIA 101 AKERN R New Edition BodyGram Plus, Pontassieve, Florence, Italy) and waist circumference was measured by an anthropometric tape to the nearest 0.001 m. All measurements were taken by the same operator before and after the study according to standard procedures [34].

### Ventilatory parameters

Resting energy expenditure (REE) and respiratory exchange ratio (RER) measurements were made by indirect calorimetry after an overnight fast (> 12 h) with subjects resting supine in comfortable thermoneutral conditions, approximately 23 °C.

The gas analysis system was used (Vmax Encore 29 System Vmax, Viasys Healthcare, Inc., Yorba Linda, CA, USA): oxygen uptake and carbon dioxide output values were measured and used to calculate REE and RER using the modified Weir equation [35] The metabolic cart was calibrated with standard gas mixture each morning. Subject were instructed to relax quietly in a dimly lit room without sleeping for 30 min and oxygen consumption (VO_2_) and dioxide production (VCO_2_) were averaged during the last 20 min for determination of respiratory exchange ratio (RER) [36], oxygen uptake was measured (mL x min^− 1^) and also normalized to body weight (mL^− 1^ x Kg^− 1^ x min^− 1^). REE: ICC = 0.99, SEM = 0.2 mL^− 1^ x Kg^− 1^ x min^− 1^. RER: ICC = 0.97, SEM = 0.02.

### Ultrasound and isometric muscle strength test

Muscle CSA of the quadriceps was measured in vivo using extended-field-of-view (EFOV) ultrasonography imaging (Mylab70, Esaote, Genoa, Italy). A 50 mm, 7.5 MHz linear array probe was used to obtain images. The 50% of the vastus lateralis length (measured from greater trochanter to the superior border of the patella) was calculated and marked with a skin-marker. The operator then positioned the probe on the medial portion of the leg, thus starting the acquisition when the medial borders of the vastus medialis had been identified. The acquisition was stopped after visualizing the lateral borders of the vastus lateralis. The pressure was kept constant during all the image acquisition.

The ultrasound scans were then analyzed using ImageJ (NIH, USA) image analysis software. Quadriceps muscle total area and vastus lateralis only area were measured following the muscle bellies borders, CSA: ICC = 0.99, SEM = 0.85 cm^2^.

Peak knee extensors muscle force (QF) of the right leg was estimated from maximal voluntary isometric contraction (MVC) at a 90° knee angle with hip fixed at 90°. Force was measured by an electrical transducer (TSD121C; BIOPAC Systems, BIOPAC Systems Inc., Goleta, CA, USA) with 1-kHz sampling frequency implemented on a custom-built chair for isometric contractions of knee extensor muscle groups. After familiarization, participants performed two 4 s-MVCs with a 2-min rest between contractions. Subjects were provided with real time visual feedback of torque production during isometric contraction. The MVC with highest QF was considered for further analysis. QF maximal value then was normalized per quadriceps muscle area to obtain force per area (N x cm^2^). MCV: ICC = 0.098, SEM = 1.8 N.

### Performance tests

#### Yo-yo intermittent recovery test level 1

The Yo-yo Intermittent Recovery Test Level 1 (YYIR1) test was developed to measure an athlete’s ability to repeatedly perform high-intensity aerobic work. Since then, it is one of the most commonly used aerobic field tests for youth and recreational athletes. It has been shown to be a valid and reliable predictor of high-intensity aerobic capacity and VO_2_max amongst athletes from various sports and competition-levels [37]. The YYIR1 focuses on an individual’s ability to repeatedly perform high-intensity aerobic work. Participants began the test from the “start line”. When instructed by the audio player, they must run towards “turn-around line 2” (this must be reached before the following beep signal) and immediately return to the “start line” before the next signal. Once “start line” is reached, participants then have a 10-s recovery period in which they must jog from “start line” towards “turn-around line 1”, and then back to “start line” before the commencement of the next shuttle. In this test the participants are only allowed two consecutive fail attempts before they are withdrawn from the test. That being, if the individual fails to reach “turn line 2” and back to “start line” in the allocated time, one fail is issued. If this happens a second consecutive time, then they are eliminated. Test-retest reliability for Yo-yo intermittent test obtained in our setting was consistent with previous findings: ICCr: Yo-yo test: 0.90, SEM: 1.3 m [38].

#### Counter movement jump test CMJ

Counter movement jump test (CMJ) was performed on a contact mat (Ergojump- Bosco system, srl, S Rufina di Cittaducale, Rieti, Italia), that allowed the measurements of height of jump, time of flight and time of contact. The CMJ starting from standing position, then subjects were instructed to perform a rapid downward movement to about 90° of knee flexion immediately followed by an upward movement. The subjects were requested to jump as high as possible. CMJ was performed three times with two minutes rest between each trial. The best performance was retained and included in the test. Test-retest reliability for CMJ obtained in our setting was consistent with previous findings: ICCr: CMJ 0.99, SEM: 0.95 cm [39].

### Statistical analysis

Results are presented as mean and standard deviation (SD). An independent samples t test was used to test baseline differences between groups. The two-way repeated-measures ANOVA was performed, with two levels by time (pre- and post-test) and considering groups (KD, WD) as inter-subject factor, in order to assess differences between groups over the course of the study (Graphpad Prism version n 4.00 for Mac, GraphPad software, San Diego, CA, USA and JASP http://www.jasp-stats.org). All differences were considered significant at *P* < 0.05 (95% CI). Post-hoc analyses were performed using the Bonferroni test. In addition, effect size (ES) calculation was done with Cohen’s *d*, as a standardized measurement based on SD differences; while d = 0.2 was considered a small affect, d = 0.5 was medium effect and d = 0.8 was a large effect, it is used as a guide for substantive significance. The normal Gaussian distribution of the data was verified by the Shapiro-Wilk test. An *unpaired t-test* with Welch’s correction were performed when appropriate.

## Results

### Dietary nutrition intake

There were no differences in dietary nutrient intakes between groups at baseline. Subjects adhered very well with the given instructions for both diet interventions according to analysis of diets records (3 days food-diary before the study and 7 days food-diary during the study). During the diet interventions, as planned, carbohydrate intake was significantly lower and fat intake significantly higher in the KD. Total dietary energy intake was reduced during both diet without significant difference (KD = 1.984 ± 340Kcal/day; WD = 1.752 ± 320Kcal/day. Importantly, protein intake, calculated both as energy percentage and grams of protein per kilogram of body weight, was similar in the two groups (Table 3).

**Table 3:** Daily intake of dietary energy and nutrients at baseline and during ketogenic diet (KD) and Western Diet (WD)

	KD PRE	KD POST	% changes	WD PRE	WD POST	% changes	2 Way ANOVA Time*Diet	Main Time effect	Main diet effect
Total (Kcal/day)	2356 ± 450	1984 ± 430	−15.78	2146 ± 230	1752 ± 320	−18.35	n.s.	< 0.001	n.s.
CHO (g/day)	350 ± 66	22 ± 5	− 93.71	363 ± 34	220 ± 56	−39.39	**< 0.0001**	< 0.0001	< 0.0001
PRO (g/day)	105 ± 20	130 ± 25	+ 23.81	121 ± 23	129 ± 28	+.6.61	n.s	n.s.	n.s.
FAT (g/day)	107 ± 20	132 ± 27	+ 23.36	110 ± 16	38 ± 10	−65.45	n.s.	< 0.001	n.s.
CHO (%)	49 ± 6	9 ± 3	−81.63	51 ± 4	51 ± 4	0	**< 0.0001**	< 0.0001	< 0.0001
PRO (%)	15 ± 3	28 ± 4	+ 86.66	14 ± 6	28 ± 3	+ 100	n.s.	< 0.0001	n.s.
FAT (%)	35 ± 4	64 ± 3	+ 82.85	33 ± 2	20 ± 8	−39.39	**< 0.0001**	< 0.0001	< 0.0001
PRO (g/kg bw/day)	1.37 ± 0.5	1.85 ± 0.3	+ 35.03	1.59 ± 0.4	1.83 ± 0.2	+ 15.09	n.s.	=0.0098	n.s.
Saturated fat (g)	35 ± 10	45 ± 12	+ 28.57	36 ± 4	15 ± 3	− 58.33	**< 0.0001**	n.s.	< 0.0001
Monounsaturated fat (g)	28 ± 6	49 ± 16	+ 75	27 ± 5	9 ± 5	−66.66	**< 0.0001**	n.s.	< 0.0001
Polyunsaturated fat (g)	16 ± 3	21 ± 5	+ 23.80	16 ± 9	5 ± 2	− 68.75	**=0.0003**	n.s	=0.0003
Cholesterol (mg)	304 ± 101	720 ± 4187	+ 136.84	303 ± 98	167 ± 65	− 44.88	**< 0.0001**	=0.0029	< 0.0001
Fibers (g)	13 ± 2	10 ± 3	−23.07	11 ± 9	15 ± 4	+ 36.36	n.s	n.s.	n.s.

Values are mean ± SD, Analysis performed on 3 days of diet records during baseline (habitual diet) and 7 days during the KD and WD

All subjects achieved the full ketogenic metabolic adjustment as indicated by color changes on the urinary reagent strips (pink-violet i.e. about 1.5 mmol/L KBs) and by capillary blood ketones levels (mean 1.3 ± 0.4 mmol/L throughout the 4 weeks). These data indicate a good compliance to the carbohydrate restriction (Fig. 2).

### Body composition, resting energy expenditure, respiratory exchange ratio, cross sectional area, strength test, performance tests

**Fig. 3:**
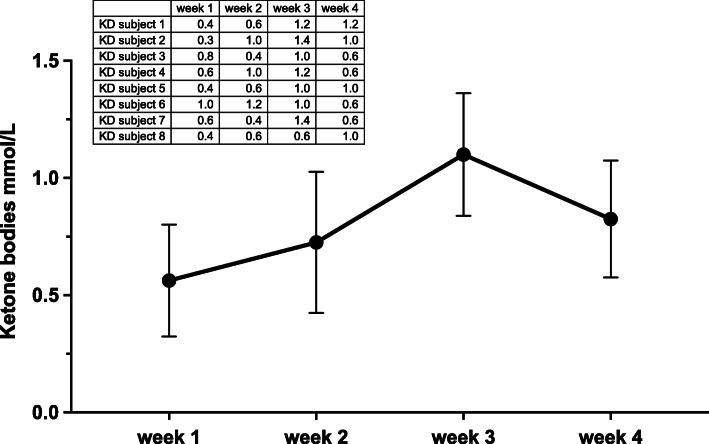
Blood ketones during the experimental period. Data are shown as mean and SD

The reduction of body fat (−1.55 Kg KD vs −0.92 Kg WD; time *diet interaction *p = 0.0359*), visceral adipose tissue VAT (− 63 g KD vs −27 g WD; time *diet interaction *p = 0.0018*), waist circumference (−4.19 cm KD vs − 1.38 cm WD; time *diet interaction *p = 0.0185*), extra cellular water (− 3.43% KD vs 0.03% WD; time *diet interaction *p = 0.0060*) were significantly greater in KD group than in WD group. Body weight decreased significantly in both groups without significant differences between groups.. Soft lean tissue mass was substantially maintained constant in both groups, as well as all hydration parameters: total body water (TBW), intracellular water (ICW), extracellular water (ECW). Moreover, no significant changes were detected in dry lean soft tissue (DLST) calculated as lean soft tissue (LST) minus TBW, and in LST hydration calculated as TBW/LST [40]. No differences were detected in appendicular nor in trunk LST measured by DXA. Diastolic blood pressure decreased significantly in both group (with a greater Δ pre vs post of 6 mmHg in KD, ES: Cohen’s *d*: 1.07, simple main effect KD; P_bonf_ < 0.001). Quadriceps muscle area and maximal strength were maintained in both groups. There were no changes for absolute and relative (Kcal/Kg) REE in both groups even though KD showed a greater Δ pre vs post difference = 1.32 Kcal/Kgbw/day, ES: Cohen’s *d*: 0.6, simple main effect KD; P_bonf_ < 0.001), whilst RER decreased significantly in KD (time *diet interaction *p = 0.0008*), further indicating a good compliance to KD diet. (Fig. 3) Yo-yo intermittent test and CMJ improved significantly (*p < 0.001*) in both groups without differences between groups.

## Discussion

To the best of our knowledge, this is the first study investigating the effects of a KD on performance in team sport, like soccer. Although many strategies are available for athletes aiming to reduce weight, the majority of those are not without risks and are not universally effective for all athletes. In our study, a team of semi-professional soccer players underwent a significant reduction in body weight, body fat mass, waist circumference, visceral adipose tissues (VAT) and extra cellular water (ECW) without negative effects on strength, power and muscle mass. Our data, in contrast to other studies [16, 18], showed no deleterious effects of KD on sport performance nor an improvement [19, 21]. Substantially our data suggest no effects of a KD on soccer-related performance tests. These conflicting results could be explained by several factors:

1. necessity to keep diet at least for 5–6 days, that is the time needed for *keto-adaptation* (as suggested in a recent paper [41];
2. electrolytes supplementation;
3. adequate protein intake in terms of quantity, quality and timing;
4. adequate hydration [12].

In our study the ketogenic phase was kept for 30 days, the duration of the diet was chosed on the bases of previous published researches [16, 18, 42–44]. As suggested by Burke and colleagues 5–6 days are enough to reach an adaptation [41] and could be defined as brief or short-term adaptation whilst 30 days could be defined as medium KD adaptation [41, 45] Adequate supplementation containing sodium and potassium in a form of plant extract, and magnesium was included. This aspect allowed to keep an healthy electrolytic balance with an adequate intake of minerals and vitamins, which is really essentials to preserve the functions of tissues [28]. It is known that micronutrients play an important role in energy production, hemoglobin synthesis, bone health, immune system and protection from oxidative stress [46]. Actually, these supplementations are not fundamental for athletes who eat an adequate quantity and good quality of foods, however, during period of low-caloric diet or unbalanced diet, such as ketogenic diet, a supplementation with minerals and vitamins may be useful to improve nutritional status and athletic performance [46]. In our study the athletes enrolled in the KD group were able to maintain their lean soft tissue. One of the reasons for this result may rely on the adequate amount of protein intake: indeed, we calculated the protein intake of the subjects according to the last evidence issued from International Society of Sport Nutrition (ISSN) [32]. Protein intake was distributed equally throughout the day (every 3–4 h): KD was provided with high protein substitutive meals (RKP), whilst WD ate high protein foods as cottage cheese, egg white, bresaola or whey protein.

Pre-sleep casein protein intake (30–40 g) was provided in both group after training evening session, as indicated by ISSN [32]. Moreover, considering that in sports field the prevention of loss of skeletal muscle during acute inflammation is fundamental, KD could be a tool to reduce inflammation [30, 47] and therefore preserve muscle mass [48]. Lastly, both groups were provided of an adequate intake of water, in order to avoid dehydration, a condition that has detrimental effects and lead to a great reduction in performance. The maintenance of strength and power performance deserves close attention. It has already been demonstrated that a 30-days of KD did not negatively affect explosive and strength performance in a group of high-level gymnasts [19], and, these positive result reflected the high intake of proteins, (~ 40% daily intake (130 ± 25 g x day ^− 1^)). In this study, strength and lean soft tissue were maintained even though the daily intake of protein was lower: we provided 1.8 g x Kg^− 1^ x body weight^− 1^ (~ 25–30% protein daily intake) in both groups. The fundamental point is that an inadequate protein intake would be likely to negatively affect performance. Even with this amount of protein though, the players showed a decrease in body mass and a maintenance of muscle mass, as a result of the well-known “*muscle- sparing effect*”, which occurs after a few days of ketosis [12]. During ketosis, the use of KBs and FFAs for energy production reduces gluconeogenesis that is related to an increased muscle protein catabolism and therefore, preserves muscle mass. Moreover, the relative increase of dietary amino-acid intake stimulates protein synthesis effect via mTOR signaling pathways [49] and it has been proposed as key factor for the preservation of lean soft tissue during KD [50] together with the anticatabolic effect in skeletal muscle given by the pleiotropic effects of ketone bodies on gene expression in muscle mass, inflammation and oxidative stress [51].. Recent studies have shown a preservation of strength in individuals on low carbohydrate diets [21, 52–54], nevertheless, dietary strategies involving carbohydrate restriction have been considered potentially able to compromise strength and power performance on longer term, as a result of diet-induced glycogen depletion [55]. The restoration of muscle glycogen by means of carbohydrate ingestion is obviously important for athletes and should not be neglected. On the other hand, KD may interfere with some muscle molecular (IGF-1, mTOR, AKT etc.) [50] and hormonal mechanisms (during the ketogenic period insulin, a powerful anabolic hormone, remains at very low levels, around 7 mU/L) related to skeletal muscle hypertrophy processes. The net balance, as suggested by recent research, seems to be the maintaining of muscle mass but a blunted hypertrophy response [54]. For athletes, the nutrients intake strategy should be tailored to the functional needs of the particular sport and, perhaps even more specifically, to the particular positional requirements within a sport and the individual needs of the athlete. One of the most striking result of the study was the ability of players to maintain their level of training and performance. This is in contradiction with the results of studies showed a decreased in performance after 10 weeks [56], 3 weeks [16], or 7 days or less of carbohydrate restricted diets [57]. This may be explained by the different subjects we investigated, indeed, for the first time, athletes of team sport were studied under a KD protocol, whilst previous studies studied mainly endurance athletes or strength/power athletes. Moreover, it should be noticed that the increasing in the Yo-Yo and CMJ in both groups from pre to post intervention, may be reasonably due to the reduction of total body mass and to the maintenance of lean soft tissue (as showed by DXA, CSA and strength) which, in turn, has improved the power/body weight ratio [19, 58]. As regard weight loss, the evidences supporting it, are certainly strong [59] and as described previously, many factors seem to be involved [60]. One suggested reason for a greater weight loss during a KD is its anorexigenic effects and hence, a reduction of daily energy intake; this was not the case of our study whereas energy intake was equivalent between the two groups. Other candidate for the greater fat loss is the increase in REE. As a matter of fact the question is still under debate: there are data suggesting that the fat loss induced by a ketogenic diet relies only on calorie deficit [61] whilst other researchers claim that KD induces an increase of REE [62, 63]. This metabolic advantage may occur during a KD due to the demand on protein turnover for gluconeogenesis, greater thermogenic effect of protein and loss of energy as heat, and/or excretion of energy in the form of ketones via urine, feces, and/or sweat. Notably, we reported no significant effect of time * diet interaction in REE (in absolute terms and weight-adjusted), after 30 days of KD. It has to be underline, however, that calculating the simple main effect for diet in KD group showed a significative result in adjusted REE (P_bonf_ < 0.001 with an ES Cohen’s *d*: 0.6). Moreover our data support the idea that an adequate intake of protein facilitates weight loss, in part, by preserving the basal metabolic rate. It is known that fat free mass is the major determinant of REE [64] and, in our study, it has been maintained in both groups. As previously pointed out, the body has a great capacity to adapt substrate oxidation to substrate intake after approximately 1 week of carbohydrate and fats, in our study fat oxidation increased, as an adaptation to the high-fat intake, typical of KD. RER decreased dramatically reflecting both the reduction in lipid synthesis and increased lipolysis mechanisms and an increase in fat metabolism for energy use. Considering the isocaloric diet, the same training protocols, and the absence of significative difference of REE’s change between groups, the measured greater fat loss in the KD deserves few more words. Our data confirmed previous findings from our group [36] and other groups [65] about the lack of a significant increase of REE during short term KD compared to a WD in training individual. However, in our study, REE in KD group showed a greater ES compared to WD. This little REE increase (1.32 Kcal/Kg bw/day; 5.47%) may explain findings from other researchers showing a significant effect of KD on REE and thus a greater weight loss during more long intervention studies [66]. Caloric intake showed no significant difference between the two groups (as showed in Table 4), even though the total dietary energy intake was slightly higher (although not significant) during KD (1.984 ± 340Kcal/day) compared to WD (1.752 ± 320Kcal/day). One another possible explanation of the greater fat loss in the KD, apart from the little increase of REE, may rely to an increased spontaneous physical activity during the day as suggested by Hall and colleagues “*… such outpatient weight loss diets may lead to greater body fat loss because of decreased energy intake and/or increased physical activity”* [67] that we unfortunately didn’t measured.

**Table 4:** Body composition, metabolic and performance values. Data are reported as mean plus SD and percentage of change

	KD PRE	KD POST	% changes	WD PRE	WD POST	% changes	2 Way ANOVA Time*Diet	Main Time effect	Main diet effect
Body Weight (Kg)	78.19 ± 11.74	73.98 ± 9.40	−5.12	76.15 ± 12.03	73.76 ± 10.13	−2.87	n.s.	< 0.001	n.s.
FM (Kg)	19.47 ± 4.07	17.92 ± 3.81	−7.93	18.88 ± 6.67	17.96 ± 6.30	−4-92	**0.036**	< 0.001	n.s.
VAT (g)	388 ± 66	325 ± 54	−16.03	355 ± 104	328 ± 101	−7.99	**0.0018**	< 0.0001	n.s.
TRUNK fat (%)	25.73 ± 3.90	24.04 ± 3.79	−6.63	24.45 ± 4.57	23.18 ± 4.37	−5.21	n.s.	< 0.001	n.s.
LST (Kg)	57.4 ± 7.10	56.9 ± 7.01	−0.87	56.21 ± 5.94	56.019 ± 5.72	- 0.34	n.s.	< 0.001	n.s.
pHa (°)	7.4 ± 0.64	7.8 ± 0.66	+ 5.63	7.32 ± 0.39	7.31 ± 0.45	−0.17	**0.003**	=0.005	n.s.
ECW (%)	40.11 ± 2.25	38.68 ± 2.10	−3.56	40.35 ± 1.22	40.38 ± 1.79	+ 0.05	**0.0060**	=0.008	n.s.
ECW (L)	19.93 ± 3.39	18.99 ± 2.63	−4.26	19.75 ± 2.96	19.58 ± 2.97	−0.91	n.s.	=0.017	n.s.
TBW (L)	49.79 ± 6.43	48.80 ± 5.39	−1.76	48.84 ± 6.55	48.31 ± 6.47	−1	n.s.	n.s.	n.s
ICW (L)	29.8 ± 3.51	29.78 ± 3.33	+ 0.1	29.49 ± 3.69	29.15 ± 3.74	−1.03	n.s.	n.s.	n.s.
DLST (Kg)	7.58 ± 1.85	8.09 ± 2.08	+ 7.28	6.96 ± 1.68	7.65 ± 1.98	+ 16.75	n.s.	n.s.	n.s.
LST Hydr %	86.72 ± 2.68	85.91 ± 2.69	−0.9	86.68 ± 2.95	86.08 ± 3.56	− 0.67	n.s.	n.s	n.s.
LST L arm (Kg)	3.25 ± 0.55	3.33 ± 0.47	+ 2.82	3.25 ± 0.4	3.39 ± 0.61	+ 4.53	n.s.	n.s	n.s.
LST R arm (Kg)	3.2 ± 0.4	3.19 ± 0.39	−0.33	3.28 ± 0.54	3.27 ± 0.4	−0.38	n.s.	n.s	n.s.
LST L leg (Kg)	9.31 ± 1.66	9.43 ± 1.5	+ 2.58	9.61 ± 1.22	9.04 ± 1.26	−5.67	n.s.	n.s	n.s.
LST R leg (Kg)	9.33 ± 1.4	10.4 ± 1.77	+ 8.15	9.74 ± 1.24	9.25 ± 1.32	−4.79	n.s.	n.s	n.s.
LST Trunk (Kg)	28.04 ± 4.49	26.38 ± 2.99	−5.3	26.61 ± 2.78	27.08 ± 3.19	+ 1.91	n.s.	n.s	n.s.
DBP	77.5 ± 4.10	72.88 ± 4.51	−6.0	78.8 ± 6.7	75.88 ± 5.93	−3.71	n.s.	< 0.001	n.s.
Waist circumference (cm)	86.75 ± 4.97	82.56 ± 3.61	−4.74	83.63 ± 8.66	82.25 ± 6.86	−1.48	**0.0185**	< 0.001	n.s.
CSA (cm2)	71.83 ± 8.32	72.20 ± 6.53	+ 0.87	71.05 ± 9.88	71.29 ± 9.247	−0.53	n.s	n.s.	n.s.
MCV (N)	628.9 ± 163.3	617.3 ± 150.2	− 0.63	621.3 ± 99.11	596.0 ± 95.56	−3.63	n.s	n.s.	n.s.
Strenght/CSA	8.754 ± 1.966	8.515 ± 1.628	−1.04	8.794 ± 1.220	8.472 ± 1.697	−4.19	n.s.	n.s.	n.s.
RER	0.87 ± 0.08	0.74 ± 0.04	−14.18	0.85 ± 0.04	0.83 ± 0.03	−2.85	**0.0008**	< 0.001	n.s.
REE (Kcal/day)	1940 ± 138.9	1939 ± 137	=	1916 ± 140.8	1917 ± 136.3	+ 0.05	n.s	n.s.	n.s.
REE (Kcal/Kg bw/day)	23.4 ± 0.8	23.3 ± 0.8	+ 5.47	22.3 ± 1.0	22.4 ± 0.8	+ 3.05	n.s	< 0.001	n.s.
Yo-yo (m)	880.4 ± 244.8	1123 ± 266.8	+ 28.04	683.0 ± 388.1	911.1 ± 378.5	+ 44.62	n.s	< 0.001	n.s.
CMJ (cm)	40.4 ± 6.5	43.6 ± 6	+ 8.52	37.3 ± 2.9	38.6 ± 04.1	+ 3.60	n.s	< 0.001	n.s.

Values are mean ± SD

*n.s.* not statistically different (*p* > 0.05)

The percentage of change was calculate through the following formula [(initial value/final value)/initial value]*100. *FM* fat mass, *VAT* visceral adipose tissue, *LST* lean soft tissue, pHa, *ECW* extra cellular water, *TBW* total body water, *ICW* intracellular water, *DLST* dry lean soft tissue, *LST Hydr* lean soft tissue hydration, *DBP* blood pressure, *CSA* cross sectional area, *MCV* maximal voluntary contraction, *RER* respiratory exchange ratio, *REE* resting energy expenditure, *CMJ* counter movement jump, *mmHg* millimeters of Mercury, *L* left, *R* right. DLST was calculated as LST-TBW; LST Hydr was calculated as TBW/LST

Regarding VAT, the majority of the published researches on KD and VAT were on persons with obesity [68–73], whilst only one previous study investigate the effect of KD on VAT in trained males [21]. Our data confirm the KD exerts a positive effect on VAT not only in persons with obesity but also in athletes.

VAT accumulation is associated with multiple cardiovascular disease (CVD) risk factors, including hypertension, impaired fasting glucose, type 2 diabetes, metabolic syndrome and low-grade chronic inflammation [74]. In athletes the reduction of the latest is of paramount importance to reduce injury risk and to improve recovery [75].

Some limits need to be considered. With regard to body composition’s analysis, a consideration should be done: as emerged from our study, both groups lost a significant amount of visceral fat measured by DXA, however, it has to be considered that DXA is an indirect approach with potential errors due to the fluid changes and hydration status of individuals [76]. For this reason, we checked hydration values demonstrating no significant changes in LST hydration.

Another limit of our research is the low sample number due to the common problem of recruiting athletes playing in sport team for experimental protocol during the competitive season. To minimize the burden on subjects, tests were performed at only at 2 time points, (beginning and end of the study), thus ketonemia was measured twice a week. Further limit could be the absence of blood exams such as pro-inflammatory cytokines; we decided to not perform hematological exams to increase adherence of athletes and in consideration of the fact that many papers have been already published about the effects of KDs on these variables.

Finally, since adherence to KD may be hard to be maintained for long periods due to the lack of some basic foods (i.e.: pasta, rice, sweets) and extra-supplements may be not always available for athletes, a specific and more accurate protocol based on convenience foods could be potentially developed in order to facilitate adherence to the KD. Moreover, a ketogenic diet can be vegetarian or vegan, with plant-based fats (i.e.., avocado, nuts, seeds, coconut, olive oil), proteins (i.e., tofu, tempeh, seitan, pea protein, veggie bacon), nonstarchy vegetables, and limited amounts of low-sugar fruits (i.e.: berries, lime, lemon, kiwis). This kind of “flexibility” allows practitioners to targeting and personalizing dietary choice on a KD. A fundamental point that can be also considered is that subjects in KD received specific plant extracts in a minimum dosage to increase the daily intake of fluid, however, the dosage was very low and could not be counted as supplements able to induce some extra effects.

## Conclusions

A common objective for many soccer players is to lose body fat while gaining or maintaining muscle mass, strength and power, although the time periods for such gains and losses are infrequent and often short.

There are several options available to lose fat. Regarding the well-known and discussed principle of energy balance, one option is to reduce energy intake by up 1000Kcal/day per week, but, this method may take longer to achieve weight loss goals and when energy intake is restricted, it is important to acknowledge the corresponding decrease in protein ingestion. An inadequate protein ingestion leads to negative effects on athletic performance, mood, perception of fatigue and decrease in lean soft tissue. Current protein intake guidelines for athletes suggest, for an average individual of 70 Kg of body weight, around 120 g of protein adequately distributed throughout the day [32]. In our study, we demonstrate that KD could be a safe and feasible strategy to lose fat mass in a short term, without impairing strength, power and muscle mass in a team sport like soccer.

Additionally, the greater reduction in VAT during KD in athletes represents a novel significant finding that deserve further investigation in higher categories soccer athletes. When the goal is a rapid weight loss reduction, coaches and athletes should consider the use of KD as a feasible and safe tool also in team sports.

## Data Availability

The datasets used and/or analysed during the current study are available from the corresponding author on reasonable request.
